# Manufacturing human mesenchymal stem cells at clinical scale: process and regulatory challenges

**DOI:** 10.1007/s00253-018-8912-x

**Published:** 2018-03-22

**Authors:** Valentin Jossen, Christian van den Bos, Regine Eibl, Dieter Eibl

**Affiliations:** 1 Institute of Chemistry and Biotechnology, Zurich University of Applied Sciences, 8820 Wädenswil, Switzerland; 2 Mares Advanced Therapies, 48268 Greven, Germany

**Keywords:** Human mesenchymal stem cells, Single-use devices, Microcarrier, Advanced therapeutic medicinal product, Good manufacturing practice, Allogeneic

## Abstract

Human mesenchymal stem cell (hMSC)-based therapies are of increasing interest in the field of regenerative medicine. As economic considerations have shown, allogeneic therapy seems to be the most cost-effective method. Standardized procedures based on instrumented single-use bioreactors have been shown to provide billion of cells with consistent product quality and to be superior to traditional expansions in planar cultivation systems. Furthermore, under consideration of the complex nature and requirements of allogeneic hMSC-therapeutics, a new equipment for downstream processing (DSP) was successfully evaluated. This mini-review summarizes both the current state of the hMSC production process and the challenges which have to be taken into account when efficiently producing hMSCs for the clinical scale. Special emphasis is placed on the upstream processing (USP) and DSP operations which cover expansion, harvesting, detachment, separation, washing and concentration steps, and the regulatory demands.

## Introduction

There is an increasing interest in human mesenchymal stem cell (hMSC)-based therapies for regenerative medicine (e.g., neurology, cardiology, immunology, orthopedics) (Aggarwal and Pittenger 2005; Ohno et al. 2012). At the beginning of November 2017, up to 190 clinical trials with hMSCs were running, of which up to 80% were within phase I and II (www.clinicaltrials.gov). These clinical trials are either done with the patient’s own cells (autologous therapies) or with cells provided by a healthy donor (allogeneic therapies) (Parton and Mason 2012; Heathman et al. 2015). Despite the large number of clinical studies, only 13 hMSC-based products have gained marketing authorization up to the present day (Heathman et al. 2015; Trounson and McDonald 2015; Chen et al. 2016; Lodge et al. 2017). Table 1 shows the nine products applicable for allogeneic therapies and the four products used for autologous transplantations. Product manufacturing takes place mainly with mesenchymal stem cells derived from human bone marrow (hBM-MSCs), followed by adipose-derived stromal/stem cells (hASCs). In addition, hMSCs derived from placental tissue, umbilical cord, cord blood, and Wharton’s jelly have become more important over the last 5 years (Heathman et al. 2015; Nordberg and Loboa 2015; Ullah et al. 2015; Chen et al. 2016).

**Table 1:** hMSC-based products with marketing authorization for allogeneic and autologous therapies

Medicinal product	Company	hMSC type	Indication	Marketing authorization
Allostem	AlloSource	Allogeneic hASC	Bone regeneration	US medical device
Cartistem	Medipost	Allogeneic UCB-MSC	Osteoarthritis	Korea
Grafix	Osiris Therapeutics	Allogeneic BM-MSC	Soft tissue defects	US medical device
Prochymal	Mesoblast	Allogeneic BM-MSC	Graft-versus-host disease	Canada and New Zealand
OsteoCel	NuVasive	Allogeneic BM-MSC	Spinal bone regeneration	US medical device
OvationOS	Osiris Therapeutics	Allogeneic BM-MSC	Bone regeneration	US medical device
TEMCELL HS	JCR Pharmaceuticals	Allogeneic BM-MSC	Graft-versus-host disease	Japan
Trinity Evolution	Orthofix	Allogeneic BM-MSC	Bone regeneration	US medical device
Trinity Elite	Orthofix	Allogeneic BM-MSC	Bone regeneration	US medical device
Hearticellgram-AMI	Pharmicell	Autologous BM-MSC	Acute myocardial infarction	Korea
Cupistem	Anterogen	Autologous hASC	Crohn’s fistula	Korea
QueenCell	Anterogen	Autologous hASC	Regeneration of subcutaneous adipose tissue	Korea
Ossron	RMS	Autologous BM-MSC	Bone regeneration	Korea

*hASC* human adipose tissue-derived stromal/stem cells, *hBM-MSC* human bone marrow-derived mesenchymal stem cells, *UCB-MSC* umbilical cord-derived mesenchymal stem cells

**Fig. 1:**
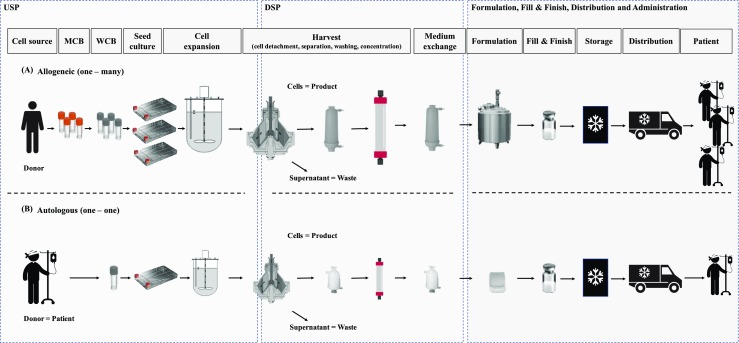
Main operations required to manufacture hMSC-based therapeutics which are used in **a** allogeneic and **b** autologous therapies

To efficiently manufacture hMSC-based products, not only must the targeted cell quantity and quality be taken into account but also the production costs. In Fig. 1a, b, the main steps involved in producing hMSC-based therapeutics for allogeneic and autologous treatments are schematically depicted. Both therapy approaches are characterized by similar manufacturing steps covering upstream processing (USP), downstream processing (DSP), formulation, and Fill&Finish operations. Typical USP operations are manufacture of the Master Cell Bank (MCB) and Working Cell Bank (WCB), seed cell production and subsequent cell expansion at L-scale. DSP steps include cell harvest, detachment of the hMSCs from their growth surface, cell separation, washing as well as concentration procedures, and medium exchange. However, before hMSCs can be administered as Advanced Therapeutic Medicinal Product (ATMP), additional formulation and Fill&Finish steps must be carried out. The main differences between allogeneic and autologous manufacturing approaches are the number of therapeutic doses generated in each batch during the cell expansion process as well as the number of patients treated. The autologous approach generates multiple small batches, with each batch yielding one or a few doses intended for one patient. In contrast, the allogeneic approach provides multiple doses for many patients. Doses are manufactured in one large batch. Due to higher cost of goods and the more crucial security and quality control described for autologous manufacturing approaches, allogeneic stem cell therapy seems to be the more commercially attractive option at present (Malik and Durdy 2015). Different economic studies have demonstrated that the USP, and in particular, the hMSC expansion, represents the main cost driver when examining the whole manufacturing process (Simaria et al. 2014; Hassan et al. 2015; Lipsitz et al. 2017). In order to achieve the high cell amounts of between 10^12^ and 10^13^ cells per batch in allogeneic hMSC manufacturing process, the manufacturer has to move away from the traditional planar cultivation platforms. Typical cell concentrations (25,000–30,000 cells/cm^2^) provided by common planar cultivation systems, which may have up to 40 layers, cannot meet the desired cell numbers and consistent quality, even at a high grade of automation and parallelization (Rowley et al. 2012; Rios and Gupta 2016; Abraham et al. 2017).

Instrumented, dynamic bioreactors operated with microcarriers have shown promising results and are meanwhile regarded as a viable alternative to planar cultivation systems in hMSC expansions (Goh et al. 2013; Rafiq et al. 2013; Santos 2014; Chen et al. 2015). A further increase in USP process safety during hMSC manufacturing was achieved by replacing reusable bioreactors with their single-use versions (Schnitzler et al. 2016; Lodge et al. 2017), which were originally designed for mammalian cell-based productions of therapeutic proteins (Kaiser et al. 2015). However, the production cells used here are presumed to be more robust and less sensitive to shear stress than hMSCs (Schnitzler et al. 2016). In addition, the culture media differ in their compositions, in particular, their supplements and generally serum (up to 20%) are present in case of hMSC expansions (Tekkatte et al. 2011; Panchalingam et al. 2015). These factors not only influence the selection of the optimum microcarrier type but also those of the optimum single-use bioreactor system and operation parameters (Tan et al. 2015, 2016). Finally, the shear stress sensitivity of the cells and the high target cell numbers and qualities affected the equipment that is today recommended for the DSP in allogeneic hMSC productions.

Our mini-review will highlight the current state of the art of allogeneic hMSC manufacturing and describe the main process and regulatory challenges for USP and DSP operations. Formulation techniques enabling the hMSCs to be frozen for shipping and storage and easily thawing at the point of clinical use will not be discussed.

## USP in hMSC productions for allogeneic therapies

### Single-use bioreactors suitable for hMSC expansion

**Fig. 2:**
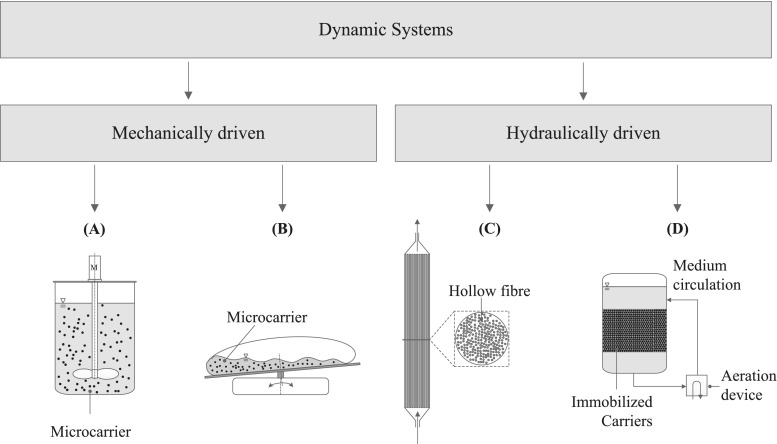
Instrumented single-use bioreactors suitable to expand hMSCs and their working principles. (A) Stirred, (B) wave-mixed, (C) hollow fiber bioreactor, and (D) fixed bed bioreactor

The majority of cultivation systems used to propagate and expand hMSCs represent single-use versions. In other words, the cultivation unit is only used once and discarded after running one batch. The single-use cultivation systems have a rigid cylindrical cultivation vessel (made from polystyrene or polycarbonate) or a flexible bag (with a polyethylene or ethylene vinyl acetate contact layer). The bag is fixed and shaped by a support container made from stainless steel and is either similar to a pillow, cube-shaped or cylindrical (Eibl et al. 2010; Eibl and Eibl 2011). Because the manufacturer provides the cultivation chambers, presterilized, sterilization, and cleaning procedures become obsolete. In addition, the risk of cross contamination is reduced which makes the hMSC expansion process safer (Kaiser et al. 2015). The disadvantages are well described for static representatives of single-use cultivation systems (planar multilayer flasks such as CELLStack, HYPERStack, and CellFactories). So, their usage is associated with inefficient mass transfer, the presence of nutrient gradients as well as inadequate process monitoring and control. Regardless, planar multilayer flasks are currently most often applied for the commercial manufacture of hMSCs (Rowley et al. 2012). The fact that dynamic single-use cultivation systems operated with microcarriers ensure easier scale-up explains the increasing interest in their usage for hMSC expansions over the past 10 years. The cultivation systems cover non-instrumented stirred (spinner flasks) (Schop et al. 2010; Kaiser et al. 2013; Jossen et al. 2016; Petry et al. 2016; Rafiq et al. 2017) as well as orbitally shaken flasks (shake flasks or Erlenmeyers) (Siddiquee and Sha 2014a) and instrumented single-use bioreactors, which are summarized in Table 2. In the case of instrumented single-use bioreactors, a distinction can be made between stirred bioreactors, wave-mixed bioreactors, hollow fiber bioreactors, and fixed bed bioreactors (Fig. 2).

**Table 2:** Instrumented, dynamic single-use bioreactors operated with microcarriers. The order of the bioreactors within each category represents no rating of the systems (alphabetically ordered)

Working principle	Scale	Manufacturer	System brand	Cell density/cell type	References
Stirred systems	Small-scale bioreactors (15 mL–0.25 L)*	Sartorius Stedim Biotech	ambr® 15/ambr® 250	4–5∙10^5^ cells/mL hBM-MSCs	Dufey et al. (2016); Nienow et al. (2016b)
		Eppendorf/DASGIP	BioBLU® 0.3c		
	Benchtop scale bioreactors	Eppendorf	CelliGen® BLU	2.7–5.3∙10^5^ cells/mL hASCs, hBM-MSCs	Milipore (2012); Cierpka et al. (2013); Siddiquee and Sha (2014a, b, 2015); Schirmaier et al. (2014); Jossen et al. (2014); Grein et al. (2016)
	(1.5–2.4 L)*	Merck	Mobius® CellReady		
		Sartorius Stedim Biotech	UniVessel® SU		
	Pilot and production scale bioreactors	Pall Life Sciences	Allegro™ STR	1.9–20∙10^5^ cells/mL hASCs, hBM-MSCs	Schirmaier et al. (2014); Rios and Gupta (2016); Lawson et al. (2017)
	(35–150 L)*	Sartorius Stedim Biotech	BIOSTAT STR®		
		Eppendorf	CelliGen® BLU		
		Thermo Scientific	HyPerforma™ SUB		
		Merck	Mobius CellReady		
		Pall Life Sciences	Nucleo®		
		GE Healthcare	Xcellerex™ XDR		
Wave-mixed	Benchtop scale (0.5–1.5 L)*	Applicon	Appiflex	1.9 ∙10^5^ cells/mL hASC, hUC-MSC	Timmins et al. (2012); Jossen et al. (2016)
		Sartorius Stedim Biotech	BIOSTAT® RM		
		Finesse	SmartRocker		
		GE Healthcare	WAVE		
		Pall Life Sciences	XRS bioreactor		
Hollow fiber	Benchtop scale	FiberCell Systems	FiberCell	10^8^–10^9^ hMSCs	Hambor (2012); Hanley et al. (2014)
	(n/a)*	Terumo BCT	Quantum Cell Expansion		
Fixed bed	Benchtop scale	Eppendorf	BioBLU® 5p	2.93 10^6^ hMSC/mL	Weber et al. (2010); Rowley et al. (2012); Tsai and Ma (2016)
	(1.0–5.0 L)*	Pall	iCellis™		

*Working volume

Stirred bioreactor versions represent the system of choice when expanding hMSCs in dynamic single-use bioreactors. But as different investigations have already shown, the biochemical engineering characterization prior to the usage of the bioreactor system allows acceptable process parameters to be predefined while avoiding cell damage and cell expansions that have failed (Hewitt et al. 2011; Kaiser et al. 2013; Schirmaier et al. 2014; Delafosse et al. 2015; Berry et al. 2016; Collignon et al. 2016; Jossen et al. 2016; Nienow et al. 2016a, c). In fact, hMSC expansion was proposed to be performed at impellers speeds, where the microcarriers are just suspended (*N*_*JS*_ *= N*_*s1*_) or below (*N*_*s1u*_) (Ibrahim and Nienow 2004; Hewitt et al. 2011; Kaiser et al. 2013). Under these conditions, a homogenous microcarrier distribution resulting in a sufficient mass transfer is achievable at minimal shear stress. Nevertheless, the acting shear stresses depend to a large extent on the performance of the bioreactor. Single-use stirred bioreactors equipped with segment blade impellers or marine impellers induce an axial oriented fluid flow which is beneficial for microcarrier-based hMSC expansions (Ibrahim and Nienow 2004; Moayeri Kashani et al. 2016). As different studies have shown, the performance of stirred single-use bioreactors for hMSC cultivations can be improved by adapting the impeller diameter, impeller off-bottom clearance or impeller blade angle (Collignon et al. 2010; Jirout and Rieger 2011; Jossen et al. 2014).

Today, instrumented stirred single-use bioreactors are commercially available at small, benchtop, and pilot/production scale. As shown in Table 2, they are used up to 150 L working volume and deliver maximum cell densities of 2 × 10^6^ hMSC/mL (Rios and Gupta 2016). Small-scale systems such as the ambr® 15 and 250 as well as the BioBLU 0.3c are suitable for medium-microcarrier screening or for scale down investigations (Szczypka et al. 2014; Dufey et al. 2016; Lipsitz et al. 2016). However, due to their small size, reliable sampling is still a challenge. Benchtop scale stirred bioreactors are preferred for first scale-up studies during the development of hMSC production processes, whereas the pilot/production scale stirred single-use bioreactors are regarded as qualified production systems. They enable GMP production of hMSCs in a class C or D room and render a class A cabinet or a class B room unnecessary (Sensebé et al. 2013). For example, Schirmaier et al. (2014) and Lawson et al. (2017) described hMSC production processes in the BIOSTAT® STR 50L and the Mobius® 50L bioreactor. Both research teams cultivated hASCs and hBM-MSCs together with microcarriers and reached maximum expansion factors of 53 (3.1 × 10^5^ hASCs/mL) under serum-reduced conditions (5% FBS) and 43 (1.9 × 10^5^ hBM-MSCs/mL) under common serum-conditions (10% FBS). The generated cell densities are sufficient to manufacture up to 177 clinical hMSC doses, assuming that 10^6^ hMSCs per kilogram body weight are applied. Rios and Gupta (2016) even reported maximum cell densities of 2.0 × 10^6^ hMSCs/mL at 50 L working volume. As also seen in our own investigations, an optimized feeding solution or/and feeding strategy can result in a process intensification as indicated by two or threefold higher cell densities (data are not shown).

It is worth mentioning that hMSC expansion processes running between 6 and 10 days usually represent batch-exchange approaches (Rios and Gupta 2016; Abraham et al. 2017). After 3 to 5 days, microcarriers are allowed to settle down before about 50% of the culture medium is replaced with fresh medium. This procedure is extremely labor intensive, and the sedimentation of the microcarriers might support the formation of large hMSC-microcarrier aggregates which are unwanted (see also “Microcarriers and their settlement in hMSC manufacturing” section). For this reason, allogeneic hMSC productions with periodical feeding and without medium exchange or with a continuous exchange of medium are underway (Rios and Gupta 2016; Abraham et al. 2017; Lawson et al. 2017). These developments presuppose microcarrier feeding as well as successful cell-to-bead and bead-to-bead transfer. For example, Leber et al. (2017) and Rafiq et al. (2017) have shown that freshly added microcarriers can be colonized by the hMSCs. However, the efficiency of the cell-to-bead and bead-to-bead transfer that is impacted by the microcarrier and its settlement still needs improvement (Takahashi et al. 2016).

### Microcarriers and their settlement in hMSC manufacturing

Microcarriers, which usually have a spherical shape, provide a high surface area to volume ratio under fully controlled process conditions in hMSC expansions realized in instrumented bioreactors. Furthermore, the growth surface can be increased within the bioreactor system to some extent, which improves the product consistency and decreases the costs due to the full use of very expensive culture media (see also “Culture medium and supplements applied in hMSC expansions” section) applied in hMSC expansions. The microcarrier type plays a key role in the cell expansion process where it is important that the critical quality attributes (CQAs) do not change. Numerous commercially available microcarrier types have already been tested in combination with hMSCs and stirred bioreactors (Table 3). The microcarrier types differ greatly in size (90–380 μm), core material (modified polystyrene, cellulose, dextran, gelatine) as well as in surface coating (collagen, fibronectin, diethylaminoethyl, triethylammonium). The core material and the surface coating do not only affect the microcarrier settlement and cell growth but also the impeller speed which is required to hold the microcarriers in suspension and to guarantee a sufficient mass transfer. Moreover, grinding events as present in certain bioreactor impeller bearings might be particularly problematic and may require subsequent purification steps downstream of cell production or, indeed, switching of bioreactor system. Rafiq et al. (2016) and Leber et al. (2017) screened different microcarrier types in small-scale bioreactors for hBM-MSCs under predefined impeller speeds (*Njs* = impeller speed required to hold the microcarriers in suspension). Both found significant differences in the cell attachment, cell growth, glucose consumption as well as the production of metabolites (e.g., lactate, ammonia) depending on the microcarrier type. They found that the hBM-MSCs have the best growth performance on collagen, Synthemax II, and ProNectin F microcarriers. This comes as no surprise, since these microcarriers are coated with collagen and fibronectin, respectively. Both coatings are components of the extracellular matrix (ECM), including the arginyl-glycyl-aspartic acid sequence which is well known for promoting cell attachment and cell growth of fastidious cells (Szczypka et al. 2014). Although, animal-free collagens are becoming more readily available, many collagen coatings are still derived from animal sources which is critical from a regulatory point of view (see also “Regulatory issues and challenges” section).

**Table 3:** Overview of microcarriers used for the expansion of hMSCs

Carrier	Manufacturer	Diameter (μm)	Material	Coating	References
Xeno-free microcarriers					
Cytodex 1	GE Healthcare	147–248	Dextran	DEAE	Takahashi et al. (2016); Rafiq et al. (2017)
Cytopore 1	GE Healthcare	200–280	Cellulose	DEAE	Takahashi et al. (2016)
Enh. Attach.	Corning	125–212	PS	CellBIND®	Rafiq et al. (2016); Leber et al. (2017)
Glass	SoloHill	125–212	PS	Silica glass	Rafiq et al. (2016); Leber et al. (2017)
Hillex®	SoloHill	160–180	Dextran	TRA	Rafiq et al. (2016, 2017)
Hillex® CT	SoloHill	90–212	PS	TRA	Rafiq et al. (2016, 2017)
Plastic	SoloHill	125–212	PS	None	Rafiq et al. (2016, 2017); Nienow et al. (2016b)
Plastic Plus	SoloHill	125–212	PS	None	Rafiq et al. (2016); Leber et al. (2017)
Star Plus	SoloHill	125–212	PS	None	Meiring et al. (2016)
Synthemax II	Corning	125–212	PS	Sy II®	Rafiq et al. (2016); Leber et al. (2017)
Mammalian protein-coated microcarriers					
Collagen	SoloHill	125–212	PS	Collagen	Nienow et al. (2016b); Leber et al. (2017); Lawson et al. (2017)
CultiSpherG	Percell Biolytica	130–380	Gelatin	None	Rafiq et al. (2016)
Cytodex 3	Ge Healthcare	141–211	Dextran	Collagen	Sart and Agathos (2016)
FACT III	SoloHill	125–212	PS	Collagen	Rafiq et al. (2016)
Recombinant protein-based microcarriers					
ProNectin F®	SoloHill	125–212	PS	Fibronectin	Jossen et al. (2016); Rafiq et al. (2016); Leber et al. (2017)

*DEAE* diethylaminoethyl, *PS* polystyrene, *Sy* synthemax, *TEA* triethylammonium

Interestingly, investigations from different research groups have demonstrated that different microcarrier surface substrates can cause a change in the phenotype, which becomes noticeable by a reduction in the expression level of different surface markers (e.g., CD90, CD105, CD166) (Keung et al. 2010; Sart et al. 2013; Rios and Gupta 2016). Moreover, recent studies have shown that the planar structure, including the material stiffness, nanotopography, and local curvature, can impact hMSC proliferation, maintenance of phenotype, and differentiation (Yim and Sheetz 2012; Zhao et al. 2014). Thus, many efforts are being made to develop new GMP-grade biodegradable microcarriers in the next years.

**Fig. 3:**
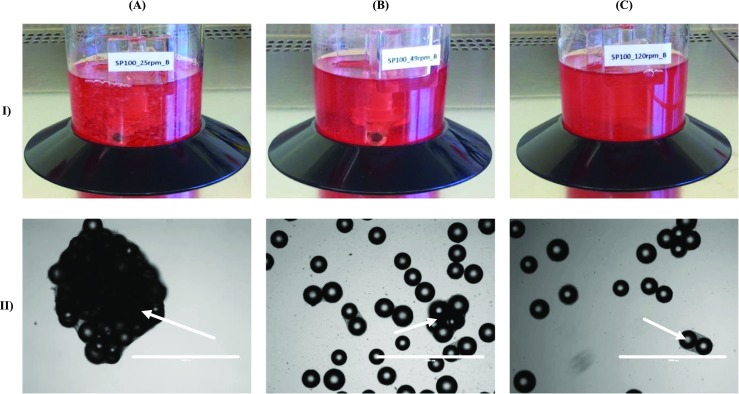
Effect of impeller speed on hMSC/MC-agglomerate formation. Macroscopic (I) and microscopic (II) images of hMSC-microcarrier-agglomerates at 25 rpm (**a**), 49 rpm (**b**), and 120 rpm (**c**) in single-use spinner flasks. The white arrows indicate the hMSC-microcarrier-agglomerates. The white scale bar corresponds to 1 mm

So far, the efficiency of microcarrier settlement covering the cell attachment and spreading and the hMSC growth is likewise dependent on the cell seeding process. In general, the cell attachment follows a Poisson distribution, where a cell-to-bead inoculation ratio of one, two, or three results in theoretical probabilities of unoccupied microcarriers of 0.365, 0.135, and 0.05 (Frauenschuh et al. 2007; Panchalingam et al. 2015). Thus, it is important to inoculate with a high cell-to-bead ratio in order to achieve a homogeneous cell distribution in which each microcarrier is occupied by at least one viable cell. Normally, a theoretical cell concentration of three to five cells per microcarrier is used. After the attachment phase, which normally takes between 4 to 20 h under static or intermitted stirred conditions, every microcarrier should have the same number of cells attached to its surface. However, in practice, this is not the case. As the investigations of our group (data not published) and Ferrari et al. (2012) have shown, a suboptimal cell seeding results in the early formation of large hMSC-microcarrier aggregates (up to 4–5 mm) which impair cell growth and stem cell characteristics. As can also be seen in Fig. 3, the aggregate size can be controlled by the impeller speed and the resulting hydrodynamic forces. This finding might be explained by the higher frequency of particle-particle interactions that facilitate an aggregation, as long as the hydrodynamic forces which can cause a cell detachment are not too high. Big aggregates may be accompanied by a decrease in the cell number which is explainable by the reduced viability in the cores of the aggregates. However, it is not yet fully understood to what extent the hMSC-microcarrier aggregate formation has an influence on the cell quality and which influence the medium composition has on the hMSC characteristics.

### Culture medium and supplements applied in hMSC expansions

Many of the conventional media used for the clinical production of hMSCs are defined basal media such as DMEM (Dulbecco’s modified Eagle medium) or *α*-MEM (Minimum Essential Media). These have to be supplemented with expensive additives such as (a) proteins which mediate adhesion to tissue culture treated plastics, (b) lipids for cellular anabolic purpose, and (c) growth factors and hormones to stimulate cellular proliferation. Even though the disadvantages of serum addition (high batch-to-batch variability, possible contamination with prions, viral and zoonotic agents, more difficult DSP and product approval, elicitation of immunologic reactions; Spees et al. 2004; Panchalingam et al. 2015) are known, the majority of hMSC culture media additionally contain between 10 and 20% (vol/vol) fetal bovine serum (FBS).

Proven alternatives to FBS are humanized media which are based on blood-derived materials such as human serum or platelet derivate (Tekkatte et al. 2011). However, humanized media are not applicable to routinely manufacture large numbers of hMSCs for allogeneic therapies due to high medium amounts and resulting costs. Moreover, the supply of high amounts of human serum is difficult to guarantee (Shahdadfar et al. 2005). Instead of human serum, human platelet lysate (hPL) was successfully applied by different groups in hMSC productions (Hemeda et al. 2014; Burnouf et al. 2016). Even though some studies have demonstrated the same proliferation potential and marker expression as in serum containing media, it is still controversially discussed whether the cells retain their immunomodulatory properties and their full differentiation capabilities (Gruber et al. 2004; Lange et al. 2007; Abdelrazik et al. 2011). Although human serum and hPL are considered to be safer than FBS, there is still a risk of human pathogens and their components being poorly characterized which may have an effect on the CQAs. This explains why there exists a high level of interest in xeno (XF) and serum-free (SF), chemically defined (CD) media for the clinical production of hMSCs.

In addition to the different XD/SF and CD media developed by academic groups (Salzig et al. 2015; Tan et al. 2016), various other formulations are on the market (e.g., MSCGM-CD from Lonza, Mesencult-XF from STEMCELL Technologies, PRIME-XV SFM from Irvine Scientific, StemPro MSC SFM XenoFree from Life Technologies, stemgro hMSC from Corning, StemXVivo® serum-free from R&D systems, SteMaxOne™ from Cell Culture Technologies, StemMACS™ XF from Miltenyi Biotec). The choice of the right SF as well as CD medium or the supplementation of an XF basal medium with the right growth factors and hormones should not be underestimated (Tekkatte et al. 2011), in particular when it is working in stirred bioreactors with microcarriers. In this case, special attention has to be given to the cell attachment efficiency (that can be lower without serum; Salzig et al. 2015; Leber et al. 2017) and the shear stress sensitivity (that is reduced without serum and requires modified process parameters or/and the addition of shear protective agents such as methylcellulose acetate). It is a fact that the expansion factors obtained so far in stirred bioreactors with XF/SF media are still significantly lower (up to 6 times) compared to those achieved in serum containing media. For example, Heathman (2015) reported a maximum expansion factor of up to 10 (hBM-MSCs) within 6 days with the PRIME-XV SF medium and a polystyrene-based microcarrier (non-porous) specifically coated with fibronectin. Tan et al. (2015) used Wharton’s Jelly-derived hMSCs in combination with the Mesencult-XF medium and a microporous microcarrier and reach an expansion factor of 6 within 6 days. These results might indicate that the use of microporous or even macroporous microcarriers is beneficial in combination with the XF/SF medium due to the higher shear protection in the porous carriers. But such microcarriers increase the complexity of the DSP. For planar expansions of hMSCs, acceptable CD media solutions are already commercially available. Our group reached comparable cell numbers (50,000–60,000 cells/cm^2^) when growing hBM-MSCs and hASCs in planar cultivation systems both with and without serum. The cultivations were executed with two CD culture media, MSCGM-CD from Lonza and UrSuppe-9 medium from the Cardio Centro Lugano. The results allow us to conclude that we need more suitable medium-microcarrier combinations for XD/SF or CD media in stirred single-use bioreactor-based expansions of hMSCs. Moreover, given these developments, one might speculate that developing and obtaining regulatory approval for serum-based products will become increasingly difficult.

Further attention must be paid to the possible appearance of extractables and leachables (chemical substances migrating from the plastic contact layer into the culture medium with cells) and to the adsorptions of medium components, all of which may result in reduced cell growth or even cell death (Mire-Sluis et al. 2016). Leachables are most dreaded for single-use systems, because they occur under real process conditions and, in the worst case, can be toxic for the patient. Until now, only leachable studies for SF animal cell-based productions of therapeutic proteins (in the majority monoclonal antibody productions with Chinese Hamster Ovary (CHO) cells) have been published (Steiger and Eibl 2013; Eibl et al. 2014; Jurkiewicz et al. 2014; Tappe et al. 2016). Blaschczock et al. (2016) also investigated the growth of hMSCs with different bioreactor bag films, but these studies were conducted with serum-containing medium. Film investigations with SF growing hMSCs are missing. They have an influence on the selection of the stirred single-use bioreactor type, because the different manufacturers apply different plastic materials and bag film layers. When growing hMSCs without serum, the early identification of critical plastic material with respect to extractables and leachables becomes more stringent. But extractable and leachable studies are more important for the DSP equipment in hMSC expansions without serum, because DSP unit operations are closer to the patient than USP unit operations. In addition, the expanded hMSCs represent the product itself which bears a higher safety risk than those of therapeutic protein productions with many purification steps.

### Downstream processing of hMSCs

#### Cell detachment and separation

The DSP of clinical hMSC manufacturing has the largest share of bottlenecks compared to USP because of the short window of time available to transfer hMSCs from the culture medium into the cryopreserved state (Abraham et al. 2017). They are the reason why the production capacity is limited to 150–200 L per batch in allogeneic hMSC productions at present. The first step after the bioreactor cultivation has been completed is the detachment of the cells from the microcarriers, which is mainly performed by proteolytic enzymes (e.g., trypsin). Here, the targets of the proteolytic enzyme are adhesion proteins on the cell surface. However, if biodegradable microcarriers are used, a mixture of two enzymes is applied, where the second enzyme (e.g., collagenase) targets the microcarrier structure. The development of such GMP-grade microcarriers is the focus of latest developments, because they significantly reduce the complexity of the subsequent separation step. Yet, in both cases, the detachment of the hMSCs from the microcarrier surface is mainly affected by the surface coating (e.g., ECM components such as collagen or fibronectin). Here, it is decisive to optimize the cell detachment procedure with respect to the maximum time the cells can be exposed to the enzyme as well as to the pH and temperature for optimal enzymatic activity. Salzig et al. (2015) tested different enzymes and ECM-components (collagen, fibronectin, laminin, vitronectin) in combination with FBS containing and SF media. They detected significant differences in the cell detachment rate (up to 40%) and cell viability (up to 20%) based on the surface coating and the enzyme used. Besides these aspects, it is also important to consider enzyme quenching or proteolytic inhibitors, especially when working with SF media after the cell detachment in order to stop the enzymatic reaction. In general, the cell detachment is mainly performed directly in the bioreactor system because the system provides precise environmental control. At the end of the cultivation, the impeller is stopped and the microcarriers are allowed to settle down on the reactor bottom before the spent medium is removed via a dip tube. At that point, the sedimentation velocity of the microcarriers or cell-microcarrier aggregates is important. Microcarriers with a density close to the medium density are beneficial in order to hold them in suspension but not so for harvesting. In the latest case, the Alternating Tangential Filtration (ATF) technology from Repligen can be used prior to cell detachment to concentrate and wash the hMSC-microcarrier suspension. XCell™ ATF modules represent effective devices to filtrate large culture volumes, while a fouling of the filtration membrane by the microcarriers is minimized (Schnitzler et al. 2016). However, it is important to define the optimum cycle times and cycle conditions to guarantee high cell recovery. During the cell detachment process, specific mechanical loads are normally applied to facilitate cell detachment. For example, Nienow et al. (2016b) used trypsin/EDTA solution together with an increased impeller speed (5x*N*_*JS*_) to improve the cell detachment from different coated and non-coated microcarriers. They worked with maximum specific power inputs of up to 2.23 W/kg and did not measure a reduction in cell viability (> 95%) or the CQAs. However, it should be kept in mind that the maximum power input and the resulting enzymatic treatment duration are influenced by the bioreactor type and its impellers.

**Fig. 4:**
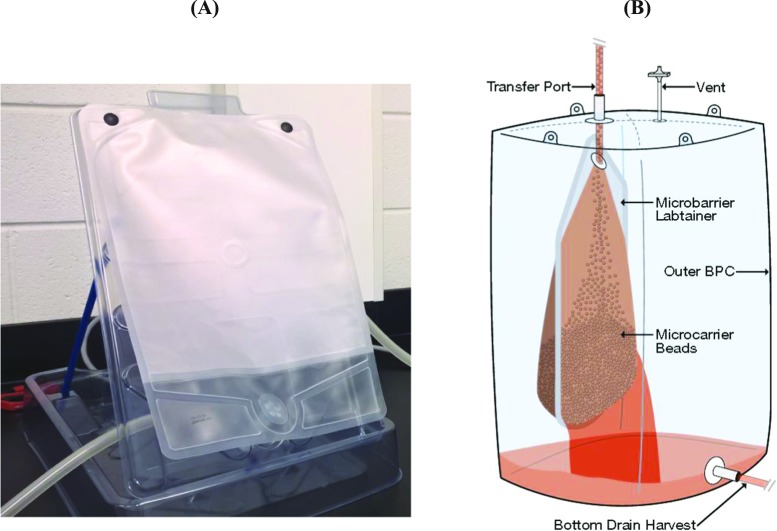
Single-use Harvestainer solution for small scale (**a**) and large scale (**b**) (with kind permission of Thermo Scientific)

After cell detachment, cells have to be separated from microcarrier beads, a process which is normally accomplished by size exclusion filtration based on size differences between detached hMSCs (~ 15–20 μm) and microcarriers (> 90 μm). At small scale, bead separation is commonly carried out using small sterile sieves such as cell strainers from Corning or blood filters from Baxter Healthcare. Our group also successfully used a sterile sieve (reusable) to harvest hMSCs from microcarriers contained in 50-L culture broth (Schirmaier et al. 2014). Schnitzler et al. (2017) applied common flow filtration device such as Steriflip® or Opticap®, which is based on a woven mesh, and separated the cells from the microcarrier beads. The single-use Harvestainer™ BioProcess Container (BPC) from Thermo Scientific is designed for small- and large-scale applications. When up to 12-L microcarrier beads have to be separated, the 3- or 12-L Harvestainer solution, consisting of a pillow-like bag and a tray, is ideal (Fig. 4a). The large-scale Harvestainer, a schematic drawing of which is shown in Fig. 4b, consists of a bag-in-bag design. The microcarriers are retained by the one or two inner Microbarrier Labtainer 25-L BPCs, while the hMSCs which are free of microcarrier beads and fragments can flow into the outer 200-L bag for further processing. Independent of the device and scale used, the target is cell recoveries and viabilities exceeding 90%. Particular emphasis has to be given to particulates (see also “Particulates” section). This is especially true when microcarrier debris may be generated by pumping out the suspension. If the resulting microcarrier debris is smaller than the filter pore size, it may contaminate the final ATMP.

#### Cell washing and concentration

Cell washing following cell detachment is required to remove any unwanted components such as FBS residues, enzyme or microcarrier debris from the cell suspension. Typically, washing is performed together with the cell concentration step needed due to the required high cell numbers (10^6^–10^7^ cells/mL) in the final formulation. Cell washing and concentration are either performed by tangential flow filtration (TFF) or by centrifugation.

TFF technology is well established in the purification of mammalian cell-based therapeutic proteins. Here, the proteins on the filtrate side are the desired product. However, the TFF technology can be adapted for clinical hMSC manufacturing by keeping the shear rate and pressure low enough. This means that the cell viability and the cell phenotype are not allowed to be affected during concentration and washing. Pattasseril et al. (2013) have reported a 60- to 100-fold concentration of hMSCs with TFF technology while maintaining cell viability and recovery at a high level. Reusable and single-use TFF systems are provided by different vendors such as EMD Millipore, Pall, Sartorius Stedim, GE Healthcare etc. However, the optimal flow rate and pressure need to be screened early in the process development in order to maximize the cell recovery.

Classical centrifugation technology is suboptimal for clinical processing of hMSCs, since the centrifugal forces compact the cells against each other and against a solid surface; cells need to be mechanically segregated subsequently, and this is likely detrimental to the cells and reduces cell recovery as well as washing efficacy. Thus, continuous flow centrifugation technology should be applied. The kSep® system from Sartorius Stedim Biotech represents a very promising device because it contains single-use tubings which are replaced after each process. hMSCs are concentrated in a fluidized bed under a continuous flow, while the clear supernatant is removed continuously. With the kSep 400 (volumetric flow rate of 114 L/h) and the kSep 6000S (volumetric flow rate of 720 L/h), the processing of high volumes within a short time becomes possible (Sartorius Stedim Biotech 2017). Independent from the technology and devices used for cell washing and concentration during the hMSC manufacture, it has to be shown once again that the concentrated hMSC culture broth contains no impurities. This also applies to the subsequent medium exchange executed with diafiltration devices and representing the last step of the DSP in hMSC manufacture (Abraham et al. 2017).

### Regulatory issues and challenges

#### Regulatory paths and agencies in the EU, USA, and Asia

In the EU, products containing living cells (and/or products of gene therapy or tissue engineering efforts) are regulated by the ATMP regulatory path (for a review, see van den Bos (2012)). Apart from common, drug-related GMP requirements, it stipulates a central procedure towards the European Market Authorization issued by the European Medicine Agency (EMA 2017). Of note, individual EU member countries offers alternative regulatory paths for products which are early in the development phase or which target exceptionally rare diseases and/or are not targeted for routine production: the so-called hospital exemption (see as follows for a review). In the USA, such products require a license from the Centre for Biologics Evaluation and Research /Food and Drug Administration, and in Asia, for example in India, must be approval by the Cellular Biology Based Therapeutic Drug Evaluation Committee advising the central regulatory agency—Central Drugs Standard Control Organization (reviewed in van den Bos et al. (2017)). Unsurprisingly, it is advisable to get in touch with the authorities as early as possible and, for example, the European Medicine Agency offers consultations for scientific advice as well as the option of having one’s preclinical work certified (Agency 2007), something which investors and/or strategic partners might appreciate. At the national level, individual member states offer various ways of providing advice, for example, the German national authority, the Paul Ehrlich Institute, offers input through its innovation office (Advice 2011).

#### Process specifics

Many ATMP products cannot be sterilized terminally which means that the entire production process is required to be aseptic. This typically requires processes to be set up in appropriate clean room cascades and/or in closed systems; it also requires risk-based assessments of high-risk process steps and their control by in-process controls; the latter rationale and execution tend to be subject for in-depth discussions with authorities.

#### Product characterization and specification

Perhaps the most pertinent issue in regard to quality and regulatory procedures is that of characterizing and specifying the product. Active Pharmaceutical Ingredients (APIs) comprising small molecules can be characterized on a per-atom basis, larger molecules such as antibodies, while clearly more complex, can still be characterized substantially. Cellular products, however, pose a significant problem in this regard.

First, therapeutic effects are sometimes observed; yet, mechanisms of action are not fully understood, complicating the choice of specifications. Second, the sheer complexity of living mammalian cells makes characterization extraordinarily difficult. While size/mass might only be a surrogate of complexity, it might help to illuminate the challenge: the molecular weight of a mammalian cell has been calculated to be 2 × 10^12^ times that of a small molecule (van den Bos et al. 2013). Thus, one might consider the comparable complexity of a mammalian cell to be very high indeed and, in turn, our ability to characterize it to be rather limited. Consequently, the process of manufacture assumes a prominent role in product characterization. Thus, changing the process from, for example, static culture to 3D culture, requires the innovator to make a case for product similarity while describing a highly complex API, something which is likely to be a point of discussion with authorities.

Mesenchymal stem cells (MSCs) have been characterized by their adherence to serum coated plastic in static cell culture by morphology (Friedenstein et al. 1970) and by expression of certain surface markers often detected by flow cytometry (Dominici et al. 2006; Bourin et al. 2013). While a combination of such markers/features has been useful to distinguish MSCs from other cells found in source tissues such as bone marrow or fat, the functional diagnostic value of such features and markers is debatable as it is difficult to associate them with particular MSC functions. This, and the absence of a single specific marker such as CD34 for hematopoietic stem cells, poses a problem in product characterization/specification. Therefore, it appears prudent to choose quantifiable markers which are also known to be directly involved in MSC functionality; secreted bioactive factors such as vascular endothelial growth factor, a factor involved in neovascularization, or enzymatic activities such as that of indolamine oxydase, an enzyme involved in the immune modulation through catabolism of tryptophan (reviewed in Mellor (2005)) have emerged as prime candidates (reviewed in van den Bos et al. (2013)). Depending on the intended product features, other markers may be chosen following the same rationale.

#### Particulates

Sometimes, cellular products are injected intravenously and hence might be considered parenteral drugs. These are required to be controlled in regard to particles, both visible and subvisible in size (see USP 787, 788, EU pharmacopeia 2.9.19). Apart from particulate matter potentially present in single-use equipment, microcarriers may, as discussed previously, contribute to the particle load of a cellular product. For any process development, it seems advisable to control for microcarrier fracturing and any potentially resulting particles. Indeed, it might be advisable to integrate this consideration into the original process development and to make low microcarrier-derived particulate levels part of the selection criteria for microcarriers.

#### Product dosing

MSCs are sometimes dosed at 1–2 × 10^6^ cells kg body weight, and this range seems to be derived empirically. A rationale for a dose might be based on desired quantifiable effects; for example, (a) which level of a bioactive factor is required in a tissue to cause a desirable effect, (b) at which level do hMSCs produce such a factor, (c) how does being in a tissue affect hMSC bioactive factor secretion levels, and (d) how many hMSCs are actually present in a tissue post application. If all of these are known, one should be able to calculate the required dose. Some of the questions, for example, (b) may be answered more easily than others, and a full set of data may not always be available. Consequently, dosing of MSCs may be based on observed therapeutic effects rather than a fully developed rationale and, as such, may be a point of discussion with authorities.

#### Product availability and storage

A campaign to manufacture larger quantities of MSCs might take 3 months including release testing. Since MSCs are often used in allogenic contexts and cryogenic storage is known to be able to preserve viability even for such fragile cells as CD34+ hematopoietic stem cells for decades (Broxmeyer et al. 2003), MSC-based products can be manufactured in advance and be retrieved from storage when needed. Assuming successful stability studies, manufacturers may thus produce in campaigns followed by potentially extended cryostorage until use. Unfortunately, sometimes, product stability appears to be substantially less than expected and this may raise quality/regulatory/economic questions. It is likely that cells which have been frozen properly, i.e., using appropriate freezing rates and suitably applied cryoprotectant, will be stable for long periods of time. If, despite this, one observes stability issues, one might consider insufficient/uneven concentrations of cryoprotectant or unintended events of elevated temperatures; these might be unlikely if cells are stored in the liquid nitrogen phase. However, it has been demonstrated that cross contamination is possible between samples stored in the liquid nitrogen phase (Bielanski et al. n.d.). Thus, for reasons of avoiding cross contamination, products are frequently stored in the vapor phase. While temperatures in the vapor phase are typically sufficiently low to warrant proper storage, the vapor phase is more susceptible to disturbance by, for example, adding or removing racks, thereby increasing the risk of accidental warming events potentially leading to a compromised product.

## Conclusions and outlook

Cell therapeutics for regenerative medicine are currently in the spotlight for researchers and manufacturers. A few hMSC-based products have already been given marketing authorization and are routinely used in clinics worldwide. However, in order to make the therapies available to a broad group of patients, interest in allogeneic therapies is particularly high. Thus, previously used planar process platforms are increasingly being replaced by instrumented single-use bioreactors which operate with microcarriers. Although different groups have already worked successfully with wave-mixed and fixed bed bioreactors, the stirred bioreactors have delivered the most promising results so far. It is believed that commercially obtainable stirred single-use bioreactors of up to 200 L culture volume are sufficient to produce hMSC-based cell therapeutic products for allogeneic therapies. However, process optimization potential still exists for XF/SF and CD culture media, which should prove cell densities of up to 1 × 10^6^ cells/mL in the future. Furthermore, culture media should be adapted to degradable GMP-grade microcarriers in order to facilitate cell growth and harvesting. The possibility of process intensification by periodical feeding and continuous medium removal has to be studied for new medium formulations. This also includes optimization of the cell-to-bead and bead-to-bead transfer and the control of the cell-microcarrier-aggregate formation. The increasing implementation of single-use devices for the USP and DSP in SF hMSC production processes may contribute to higher process and patient safety, and presupposed extractables and leachables at critical levels can be also excluded. But this requires risk analyses and extractable and leachable studies for hMSC production processes for which no recommendations yet exist. Even though a clear regulatory path for the approval of hMSC-based products does exist, certain challenges remain. One such challenge might be the problem of particulates because hMSCs-based products cannot be terminally filter sterilized. Also, from a regulatory point of view, one might speculate that developing and obtaining regulatory approval for serum-based products will become increasingly difficult in the future.

## References

[CR1] Abdelrazik H, Spaggiari GM, Chiossone L, Mretta L (2011). Mesenchymal stem cells expanded in human platelet lysate display a decreased inhibitory capacity on T- and NK-cell proliferation and function. Eur J Immunol.

[CR2] Abraham E, Gupta S, Jung S, McAfee E (2017) Bioreactor for scale-up: process control. In: Mesenchymal stem cells. pp 139–177

[CR3] Advice R (2011) The Innovation Office at the Paul-Ehrlich-Institut

[CR4] Agency EM (2007) European Medicines Agency—advanced therapy development—certification procedures for micro-, small- and medium-sized enterprises (SMEs). http://www.ema.europa.eu/ema/index.jsp?curl=pages/regulation/general/general_content_000300.jsp&mid=WC0b01ac058095e6d5

[CR5] Aggarwal S, Pittenger MF (2005). Human mesenchymal stem cells modulate allogeneic immune cell responses. Blood.

[CR6] Berry JD, Liovic P, Šutalo ID, Stewart RL, Glattauer V, Meagher L (2016). Characterisation of stresses on microcarriers in a stirred bioreactor. Appl Math Model.

[CR7] Bielanski A, Nadin-Davis S, Sapp T, Lutze-Wallace C (n.d.) Viral contamination of embryos cryopreserved in liquid nitrogen. 10.1006/cryo.1999.2227 10.1006/cryo.1999.2227 10788310

[CR8] Blaschczock K, Steiger N, Jurkiewicz E, Husemann U, Faulstich F, Eibl R, Greller G (2016). Evaluating a new film for single-use bags. BioProcess Int.

[CR9] Bourin P, Bunnell BA, Casteilla L, Dominici M, Katz AJ, March KL, Redl H, Rubin JP, Yoshimura K, Gimble JM (2013). Stromal cells from the adipose tissue-derived stromal vascular fraction and culture expanded adipose tissue-derived stromal/stem cells: a joint statement of the International Federation for Adipose Therapeutics and Science (IFATS) and the International Society for Cellular Therapy (ISCT). Cytotherapy.

[CR10] Broxmeyer HE, Srour EF, Hangoc G, Cooper S, Anderson SA, Bodine DM (2003). High-efficiency recovery of functional hematopoietic progenitor and stem cells from human cord blood cryopreserved for 15 years. Proc Natl Acad Sci U S A.

[CR11] Burnouf T, Strunk D, Koh MBC, Schallmoser K (2016). Human platelet lysate: replacing fetal bovine serum as a gold standard for human cell propagation?. Biomaterials.

[CR12] Chen AKL, Chew YK, Tan HY, Reuveny S, Oh SKW (2015). Increasing efficiency of human mesenchymal stromal cell culture by optimization of microcarrier concentration and design of medium feed. Cytotherapy.

[CR13] Chen YS, Chen YA, Tsai PH, Chen CP, Shaw SW, Hsuan Y (2016). Mesenchymal stem cell: considerations for manufacturing and clinical trials on cell therapy product. Int J Stem Cell Res Ther.

[CR14] Cierpka K, Elseberg CL, Niss K, Kassem M, Salzig D, Czermak P (2013). hMSC production in disposable bioreactors with regards to GMP and PAT. Chemie Ing Tech.

[CR15] Collignon M-L, Delafosse A, Crine M, Toye D (2010). Axial impeller selection for anchorage dependent animal cell culture in stirred bioreactors: methodology based on the impeller comparison at just-suspended speed of rotation. Chem Eng Sci.

[CR16] Collignon ML, Delafosse A, Calvo S, Martin C, Marc A, Toye D, Olmos E (2016). Large-Eddy Simulations of microcarrier exposure to potentially damaging eddies inside mini-bioreactors. Biochem Eng J.

[CR17] Delafosse A, Collignon M-L, Marc A, Toye D, Olmos E (2015). Revisiting the determination of hydromechanical stresses encountered by microcarriers in stem cell culture bioreactors. BMC Proc.

[CR18] Dominici M, Le Blanc K, Mueller I, Slaper-Cortenbach I, Marini F, Krause D, Deans R, Keating A, Prockop D, Horwitz E (2006). Minimal criteria for defining multipotent mesenchymal stromal cells. The International Society for Cellular Therapy position statement. Cytotherapy.

[CR19] Dufey V, Tacheny A, Art M, Becken U, De Longueville F (2016). Expansion of human bone marrow-derived mesenchymal stem cells in BioBLU 0.3c single-use bioreactors. Appl Note.

[CR20] Eibl R, Eibl D (2011) Single-use technology in biopharmaceutical manufacture. John Wiley & Sons

[CR21] Eibl R, Kaiser S, Lombriser R, Eibl D (2010). Disposable bioreactors: the current state-of-the-art and recommended applications in biotechnology. Appl Microbiol Biotechnol.

[CR22] Eibl R, Steiger N, Fritz C, Eisenkrätzer D, Bär J, Müller D, Eibl D (2014) Recommendation for leachables studies: standardized cell culture test for the early identification of critical films for CHO cell lines in chemically defined culture media. DECHEMA, Frankfurt am Main, p 1–22

[CR23] EMA (2017) European Medicines Agency—human regulatory—pharmacovigilance. http://www.ema.europa.eu/ema/index.jsp?curl=pages/regulation/general/general_content_000258.jsp

[CR24] Ferrari C, Balandras F, Guedon E, Olmos E, Chevalot I, Marc A (2012). Limiting cell aggregation during mesenchymal stem cell expansion on microcarriers. Biotechnol Prog.

[CR25] Frauenschuh S, Reichmann E, Ibold Y, Goetz PM, Sittinger M, Ringe J (2007). A microcarrier-based cultivation system for expansion of primary mesenchymal stem cells. Biotechnol Prog.

[CR26] Friedenstein AY, Chailakhlan RK, Lalykina KS (1970). The development of fibroblast colonies of fibroblast colonies in monolayer cultures of guinea-pig bone marrow and spleen cells. Cell Tissue Kinet.

[CR27] Goh T, Zhang Z, Chen A, Reuveny S, Choolani M, Chan J, Oh S (2013). Microcarrier culture for efficient expansion and osteogenic differentiation of human fetal mesenchymal stem cells. Biores Open Access.

[CR28] Grein TA, Leber J, Blumenstock M, Petry F, Weidner T, Salzig D, Czermak P (2016). Multiphase mixing characteristics in a microcarrier-based stirred tank bioreactor suitable for human mesenchymal stem cell expansion. Process Biochem.

[CR29] Gruber R, Karreth F, Kandler B, Fuerst G, Rot A, Fischer AB (2004). Platelet-released supernatants increase migration and proliferation, and decrease osteogenic differentiation of bone marrow-derived mesenchymal progenitor cell under in vitro conditions. Platelets.

[CR30] Hambor J (2012). Bioreactor design and bioprocess controls for industrialized cell processing. BioProcess Int.

[CR31] Hanley PJ, Mei Z, Durett AG, da Graca Cabreira-Harrison M, Klis M, Li W, Zhao Y, Yang B, Parsha K, Mir O, Vahidy F, Bloom D, Rice RB, Hematti P, Savitz SI, Gee AP (2014). Efficient manufacturing of therapeutic mesenchymal stromal cells with the use of the Quantum Cell Expansion System. Cytotherapy.

[CR32] Hassan S, Simaria AS, Varadaraju H, Gupta S, Warren K, Farid SS (2015). Allogeneic cell therapy bioprocess economics and optimization: downstream processing decisions. Regen Med.

[CR33] Heathman TRJ (2015). Expansion, harvest and cryopreservation of human mesenchymal stem cells in a serum-free microcarrier process. Biotechnol Bioeng.

[CR34] Heathman TRJ, Nienow AW, McCall MJ, Coopman K, Kara B, Hewitt CJ (2015). The translation of cell-based therapies: clinical landscape and manufacturing challenges. Regen Med.

[CR35] Hemeda H, Giebel B, Wagner W (2014). Evaluation of human platelet lysate versus fetal bovine serum for culture of mesenchymal stromal cells. Cytotherapy.

[CR36] Hewitt CJ, Lee K, Nienow AW, Thomas RJ, Smith M, Thomas CR (2011). Expansion of human mesenchymal stem cells on microcarriers. Biotechnol Lett.

[CR37] Ibrahim S, Nienow AW (2004). Suspension of microcarriers for cell culture with axial flow impellers. Chem Eng Res Des.

[CR38] Jirout T, Rieger F (2011). Impeller design for mixing of suspensions. Chem Eng Res Des.

[CR39] Jossen V, Kaiser SC, Schirmaier C, Herrmann J, Tappe A, Eibl D, Siehoff A, van d BC, Eibl R (2014). Modification and qualification of a stirred single-use bioreactor for the improved expansion of human mesenchymal stem cells at benchtop scale. Pharm Bioprocess.

[CR40] Jossen V, Schirmer C, Mostafa Sindi D, Eibl R, Kraume M, Pörtner R, Eibl D (2016). Theoretical and practical issues that are relevant when scaling up hMSC microcarrier production processes. Stem Cells Int.

[CR41] Jurkiewicz E, Husemann U, Greller G, Barbaroux M, Fenge C (2014). Verification of a new biocompatible single-use film formulation with optimized additive content for multiple bioprocess applications. Biotechnol Prog.

[CR42] Kaiser S, Jossen V, Schirmaier C, Eibl D, Brill S, van den Bos C, Eibl R (2013). Fluid flow and cell proliferation of mesenchymal adipose-derived stem cells in small-scale, stirred, single-use bioreactors. Chemie Ing Tech.

[CR43] KaiserSC, EiblD, EiblR (2015) Single-use bioreactors for animal and human cells. In: Animal cell culture: cell engineering pp 445–499

[CR44] Keung AJ, Kumar S, Schaffer DV (2010). Presentation counts: Microenvironmental regulation of stem cells by biophysical and material cues. Annu Rev Cell Dev Biol.

[CR45] Lange C, Cakiroglu F, Spiess AN, Cappallo-Obermann H, Dierlamm J, Zander AR (2007). Accelerated and safe expansion of human mesenchymal stromal cells in animal serum-free medium for transplantation and regenerative medicine. J Cell Physiol.

[CR46] Lawson T, Kehoe DE, Schnitzler AC, Rapiejko PJ, Der KA, Philbrick K, Punreddy S, Rigby S, Smith R, Feng Q, Murrell JR, Rook MS (2017). Process development for expansion of human mesenchymal stromal cells in a 50 L single-use stirred tank bioreactor. Biochem Eng J.

[CR47] Leber J, Barekzai J, Blumenstock M, Pospisil B, Salzig D, Czermak P (2017). Microcarrier choice and bead-to-bead transfer for human mesenchymal stem cells in serum-containing and chemically defined media. Process Biochem.

[CR48] Lipsitz YY, Timmins NE, Zandstra PW (2016). Quality cell therapy manufacturing by design. Nat Biotechnol.

[CR49] Lipsitz YY, Milligan WD, Fitzpatrick I, Stalmeijer E, Farid SS, Tan KY, Smith D, Perry R, Carmen J, Chen A, Mooney C, Fink J (2017). A roadmap for cost-of-goods planning to guide economic production of cell therapy products. Cytotherapy.

[CR50] Lodge A, Detela G, Barry J, Ginty P, Mount N (2017) Global regulatory perspective for MSCs. In: Mesenchymal Stem Cells pp 243–287

[CR51] Malik NN, Durdy MB (2015) Cell therapy landscape. In: Translational regenerative medicine. Elsevier, pp 87–106

[CR52] Meiring A, Schneider I, Beasley S, Woods E (2016) Scalable production of human mesenchymal stem cells in a novel bioreactor using a xenogenic free culture system. In: 22nd Annual ISCT Meeting. p 1

[CR53] Mellor A (2005). Indoleamine 2,3 dioxygenase and regulation of T cell immunity. Biochem Biophys Res Commun.

[CR54] Milipore EM (2012) Scale-up of human mesenchymal stem cells on microcarriers in suspension in single-use bioreactor. Appl Note:1–8

[CR55] Mire-Sluis A, Ma S, Marcovic I (2016). Extractables and leachables: challenges and strategies in biopharmaceutical development. BioProcess Int Suppl.

[CR56] Moayeri Kashani M, Lai SH, Ibrahim S, Moradi Bargani P (2016). Design factors affecting the dynamic performance of soil suspension in an agitated, baffled tank. Chin J Chem Eng.

[CR57] Nienow AW, Coopman K, HeathmanTRJ, RafiqQA, HewittCJ (2016a) Chapter 3: bioreactor engineering fundamentals for stem cell manufacturing. In: Stem cell manufacturing. Elsevier, pp 43–75

[CR58] Nienow AW, Hewitt CJ, Heathman TRJ, Glyn VAM, Fonte GN, Hanga MP, Coopman K, Rafiq QA (2016). Agitation conditions for the culture and detachment of hMSCs from microcarriers in multiple bioreactor platforms. Biochem Eng J.

[CR59] Nienow AW, Rafiq QA, Heathman TRJ, Coopman K, Hewitt CJ (2016). Mixing theory for culture and harvest in bioreactors of human mesenchymal stem cells on microcarriers. Theor Found Chem Eng.

[CR60] Nordberg RC, Loboa EG (2015). Our fat future: translating adipose stem cell therapy. Stem Cells Transl Med.

[CR61] Ohno T, Kaneda H, Nagai Y, Fukushima M (2012). Regenerative medicine in critical limb ischemia. J Atheroscler Thromb.

[CR62] Panchalingam KM, Jung S, Rosenberg L, Behie LA (2015). Bioprocessing strategies for the large-scale production of human mesenchymal stem cells: a review. Stem Cell Res Ther.

[CR63] Parton S, Mason C (2012). A decade of cell therapy clinical trials (2000–2010). Regen Med.

[CR64] Pattasseril J, Varadaraju H, Lock L, Rowley J (2013). Downstream technology landscape for large-scale therapeutic cell processing. BioProcess Int.

[CR65] PetryF, SmithJR, LeberJ, SalzigD, CzermakP, WeissML (2016) Manufacturing of human umbilical cord mesenchymal stromal cells on microcarriers in a dynamic system for clinical use. 2016:1–33. 10.1155/2016/4834616 10.1155/2016/4834616 PMC4761675 26977155

[CR66] Rafiq QAQA, Brosnan KM, Coopman K, Nienow AW, Hewitt CJ (2013). Culture of human mesenchymal stem cells on microcarriers in a 5 L stirred-tank bioreactor. Biotechnol Lett.

[CR67] Rafiq QA, Coopman K, Nienow AW, Hewitt CJ (2016). Systematic microcarrier screening and agitated culture conditions improves human mesenchymal stem cell yield in bioreactors. Biotechnol J.

[CR68] Rafiq QA, Ruck S, Hanga M, Heathman TRJ, Coopman K, Nienow AW, Williams DJ, Hewitt CJ (2017) Qualiative and quantitative demonstration of bead-to-bead transfer with bone marrow-derived human mesenchymal stem cells on microcarriers: utilising the phenomenon to improve culture performance. Biochem Eng J. 10.1016/j.bej.2017.11.005

[CR69] Rios M, Gupta S (2016). Bioreactor manufacturing platforms for cell therapies. BioProcess Int.

[CR70] Rowley J, Abraham E, Campbell A, Brandwein H, Oh S (2012). Meeting lot-size challenges of manufacturing adherent cells for therapy. BioProcess Int Suppl.

[CR71] Salzig D, Leber J, Merkewitz K, Lange MC, Köster N, Czermak P (2015). Attachment, growth and detachment of human mesenchymal stem cells in a chemically defined medium. Stem Cells Int.

[CR72] Santos F (2014). A xenogeneic-free bioreactor system for the clinical-scale expansion of human mesenchymal stem/stromal cells. Biotechnol Bioeng.

[CR73] Sart S, Agathos SN (2016) Large-scale expansion and differentiation of mesenchymal stem cells in microcarrier-based stirred bioreactors. Methods Mol Biol:257–284. 10.1007/7651 10.1007/7651_2015_314 26892015

[CR74] Sart S, Agathos SN, Li Y (2013). Engineering stem cell fate with biochemical and biomechanical properties of microcarriers. Biotechnol Prog.

[CR75] Sartorius Stedim Biotech (2017) kSep systems (data sheet). 1–4

[CR76] Schirmaier C, Jossen V, Kaiser SC, Jüngerkes F, Brill S, Safavi-Nab A, Siehoff A, van den Bos C, Eibl D, Eibl R (2014). Scale-up of adipose tissue-derived mesenchymal stem cell production in stirred single-use bioreactors under low-serum conditions. Eng Life Sci.

[CR77] Schnitzler AC, Verma A, Kehoe DE, Jing D, Murrell JR, Der KA, Aysola M, Rapiejko PJ, Punreddy S, Rook MS (2016). Bioprocessing of human mesenchymal stem/stromal cells for therapeutic use: current technologies and challenges. Biochem Eng J.

[CR78] Schnitzler AC, Rigby S, Aysola M, Murrell J (2017) Harvest of human mesenchymal stem cells from microcarriers. BioPharm Int 2017:23–26

[CR79] Schop D, van Dijkhuizen-Radersma R, Borgart E, Janssen FW, Rozemuller H, Prins HJ, de Bruijn JD (2010). Expansion of human mesenchymal stromal cells on microcarriers: growth and metabolism. JTissue Eng Regen Med.

[CR80] Sensebé L, Gadelorge M, Fleury-Cappellesso S (2013). Production of mesenchymal stromal/stem cells according to good manufacturing practices: a review. Stem Cell Res Ther.

[CR81] Shahdadfar A, Fronsdal K, Haug T, Reinholt FP, Brinchmann JE (2005). In vitro expansion of human mesenchymal stem cells: choice of serum is a determinant of cell proliferation, differentiation, gene expression, and transcriptome stability. Stem Cells.

[CR82] Siddiquee K, Sha M (2014). Microcarrier-based expansion of adipose-derived mesenchymal stem cells in shake flasks. Bioprocess J.

[CR83] Siddiquee K, Sha M (2014). Large-scale production of human mesenchymal stem cells in BioBLU 5c single-use vessels. Appl Note.

[CR84] Siddiquee K, Sha M (2015). Billion-cell hypoxic expansion of human mesenchymal stem cells in BioBLU 5c single-use vessels. Bioprocess J.

[CR85] Simaria AS, Hassan S, Varadaraju H, Rowley J, Warren K, Vanek P, Farid SS (2014). Allogeneic cell therapy bioprocess economics and optimization: single-use cell expansion technologies. Biotechnol Bioeng.

[CR86] Spees JL, Gregory CA, Singh H, Tucker HA, Peister A, Lynch PJ (2004). Internalized antigens must be removed to prepare hypoimmunogenic mesenchymal stem cells for cell and gene therapy. Mol Ther.

[CR87] Steiger N, Eibl R (2013). Interlaboratory test for detection of cytotoxic leachables arising from single-use bags. Chemie Ing Tech.

[CR88] Szczypka M, Splan D, Woolls H, Brandwein H (2014). Single-use bioreactors and microcarriers. Bioprocess Int.

[CR89] Takahashi I, Sato K, Mera H, Wakitani S, Takagi M (2016). Effects of agitation rate on aggregation during beads-to-beads subcultivation of microcarrier culture of human mesenchymal stem cells. Cytotechnology.

[CR90] Tan KY, Teo KL, Lim JFY, Chen AKL, Reuveny S, Oh SKW (2015). Serum-free media formulations are cell line-specific and require optimization for microcarrier culture. Cytotherapy.

[CR91] Tan KY, Reuveny S, Oh SKW (2016). Recent advances in serum-free microcarrier expansion of mesenchymal stromal cells: parameters to be optimized. Biochem Biophys Res Commun.

[CR92] Tappe A, Cutting J, Hammnond M (2016). The case for a standardized assay to test suitability of single-use sytems in cell culture applications. BioProcess Int.

[CR93] Tekkatte C, Gunasingh GP, Cherian KM, Sankaranarayanan K (2011). “Humanized” stem cell culture techniques: the animal serum controversy. Stem Cells Int.

[CR94] Timmins NE, Kiel M, Günther M, Heazlewood C, Doran MR, Brooke G, Atkinson K (2012). Closed system isolation and scalable expansion of human placental mesenchymal stem cells. Biotechnol Bioeng.

[CR95] Trounson A, McDonald C (2015). Stem cell therapies in clinical trials: progress and challenges. Cell Stem Cell.

[CR96] Tsai A-C, Ma T, Turksen K (2016). Expansion of human mesenchymal stem cells in a microcarrier bioreactor. Bioreactors in stem cell biology: methods and protocols.

[CR97] UllahI, SubbaraoRB, RhoGJ (2015) Human mesenchymal stem cells—current trends and future prospective 10.1042/BSR20150025 PMC4413017 25797907

[CR98] van den Bos C (2012) Off the beaten track—regulatory changes. Eur Biopharm Rev: 32–36

[CR99] van den Bos C, Keefe R, Schirmaier C, McCaman M (2013) Therapeutic human cells: manufacture for cell therapy/regenerative medicine. In: Advances in biochemical engineering/biotechnology. pp 61–97 10.1007/10_2013_233 23934363

[CR100] van den Bos C, Walter B, Somaiah M (2017) Standing together. Eur Biopharm Rev:32–25

[CR101] Weber C, Freimark D, Pörtner R, Pino-Grace P, Pohl S, Wallrapp C, Geigle P, Czermak P (2010). Expansion of human mesenchymal stem cells in a fixed-bed bioreactor system based on non-porous glass carrier—part A: inoculation, cultivation, and cell harvest procedures. Int J Artif Organs.

[CR102] Yim EK, Sheetz MP (2012). Force-dependent cell signaling in stem cell differentiation. Stem Cell Res Ther.

[CR103] Zhao W, Li X, Liu X, Zhang N, Wen X (2014). Effects of substrate stiffness on adipogenic and osteogenic differentiation of human mesenchymal stem cells. Mater Sci Eng C.

